# Mitigating heat stress in full-time grazing dairy cows in temperate climates: The impact of indoor housing during the hottest time of day

**DOI:** 10.1016/j.vas.2025.100488

**Published:** 2025-07-16

**Authors:** Alice Pontiggia, Mirjam Holinger, Andreas Münger, Stefanie Ammer, Frigga Dohme-Meier, Nina Maria Keil

**Affiliations:** a Centre for Proper Housing of Ruminants and Pigs, Swiss Federal Food Safety and Veterinary Office, Agroscope, Route de la Tioleyre 4, 1725 Posieux, Switzerland; b Ruminant Nutrition and Emissions, Agroscope, Route de la Tioleyre 4, 1725 Posieux, Switzerland; c Department of Livestock Sciences, Research Institute of Organic Agriculture (FiBL), Ackerstrasse 113, 5070 Frick, Switzerland; d Department of Animal Sciences, University of Göttingen, Albrecht-Thaer-Weg 3, 37075 Göttingen, Germany

**Keywords:** Comprehensive climate index, Vaginal temperature, Reticular temperature, Respiration rate, Heart rate, Moderate heat stress

## Abstract

We hypothesised that bringing full-time grazing dairy cows indoors during the hottest time of summer days could reduce heat stress if barns are cooler than outdoor. A 51-day pilot study on 19 pasture-based Swiss dairy farms revealed that, with daily mean ambient temperatures between 15 and 22 °C, barns were on average 2.5 °C cooler than pasture between 1000 and 1800 h. In an experiment over two summers, 38 Holstein dairy cows were investigated in 12 experimental periods of up to three consecutive days, with a mean daily comprehensive climate index between 23.6 and 28.5 °C. Cows experienced two treatments with a cross-over after each experimental period: half of the cows stayed on pasture full time except during milking, while the other half was brought inside the barn from 1130 h until afternoon milking and was supplemented with hay. During this time, barn conditions were cooler (mean and SD −2.9 ± 3.8 °C) than those on pasture. Between 0830 and 1100 h, when all cows were on pasture, no differences in physiological and behavioural indicators were detected in relation to treatment. Between 1200 and 1430 h, cows kept inside had lower vaginal temperature, reticular temperature, heart rate and respiration rate compared with those on pasture. Cows kept inside spent less time feeding and walking, but more time ruminating and lying down. These results suggest that bringing dairy cows inside during the hottest time of summer days reduces heat stress in grazing systems if barns are cooler than outdoors.

## Highlights

• Full-time dairy grazing dairy cows are exposed to solar radiation.
• Barns can be expected to be cooler than outdoors in the afternoon of summer days.
• Cows indoors had less physiological responses to the heat load than oudoors.
• Housing cows indoors in the afternoon can be recommended to mitigate heat stress.

## 1 Introduction

Pasture-based milk production systems have some important benefits for the welfare of dairy cows. They have the potential to improve health (e.g. reduced levels of lameness, hoof pathologies, hock lesions, mastitis, uterine diseases and mortality) and improve wellbeing by allowing dairy cows to express their full behavioural repertoire (leading to e.g. benefits in terms of grazing, increased duration of lying and reduced aggression) (Arnott et al., 2017). On the other hand, exposure to high temperature and intense solar radiation can induce heat stress (Pontiggia et al., 2023, 2024) with deleterious effects on welfare, health and productivity of dairy cows on pasture even in temperate summers (Van Laer et al., 2015a, 2015b; Veissier et al., 2018). Offering shade can protect cows from solar radiation; consequently, grazing dairy cows with access to shade show lower core body temperature and respiration rate and higher milk production than animals without access to shade (Van Laer et al., 2015a, 2015b; Veissier et al., 2018).

However, when milk production is based on intensive use of pasture where rotational or strip-grazing is applied, it can be difficult to provide adequate shade structures on every single paddock. To mitigate heat stress on hot summer days, one measure in temperate regions is to bring the cows inside during the hottest time of day, i.e. in the early afternoon (Thanner et al., 2014). However, it has not yet been proven if this is an effective measure. Previous studies have revealed that environmental conditions inside the barns can vary considerably depending on the construction of the barn (VanderZaag et al., 2023). To effectively reduce heat stress, the climate conditions in the barn must be cooler than those on pasture. Cows in the barn are protected from solar radiation, but there are other parameters (especially ambient temperature, relative humidity and wind speed) that influence barn climate conditions. The comprehensive climate index (CCI) combines ambient temperature, relative humidity, solar radiation and wind speed into one indicator of heat load (Mader et al., 2010, 2011) and can be used to compare conditions on pasture and in the barn.

A reliable indicator of heat stress in dairy cows is core body temperature (Veissier et al., 2018; Pontiggia et al., 2023). Vaginal and rectal temperature measurements are both recognized as accurate methods for assessing core body temperature (Idris et al., 2021). In contrast to the rectal temperature, the vaginal temperature can be measured continuously in dairy cows, which makes its measurement applicable for continuous monitoring of heat stress. Values of core body temperature greater than 39 °C clearly indicate heat stress (Kadokawa et al., 2012; Veissier et al., 2018). Heat-stressed animals also show physiological and behavioural coping strategies to regulate their core body temperature at the set value (Becker et al., 2020). For example, to release as much body heat as possible, dairy cows increase their respiration rate (Kadzere et al., 2002) and heart rate (Jo et al., 2021). Heat dissipation is also facilitated while standing, and heat-stressed dairy cows have been shown to reduce lying time in free-stall systems (Herbut & Angrecka, 2018). To lower their metabolic heat production, heat-stressed cows also reduce their feed intake and rumination time (Kadzere et al., 2002). Reduced feed intake due to heat load exposure can lead to depression in milk yield and alterations in its content of fat, lactose and protein (Van Laer et al. 2015a).

The objective of the present study was to evaluate if under conditions of full-time grazing bringing dairy cows inside during the hottest time of summer days is an effective measure to reduce heat stress under temperate climate conditions. In a pilot study, we surveyed the climate conditions inside and outside the barns of 19 dairy farms with pasture-based milk production in Switzerland to see if conditions in the barn cooler than outside can be expected in farming practice during the hottest time of day. In a following experimental study, we analysed to which extent bringing dairy cows inside the barn during the hottest time of day mitigates heat stress when cooler conditions are provided. We expected that cows kept inside the barn would show lower vaginal temperatures, reticular temperatures, respiration rates and heart rates than cows staying on pasture. We also expected changes in feeding behaviour and activity due to the differing heat load, housing conditions and the feed supplementation in the barn.

## 2 Materials and methods

### 2.1 Pilot study: Climate conditions in barns of farms with pasture-based milk production

#### 2.1.1 Data collection

Nineteen Swiss dairy farms were included in the pilot study based on the criteria that cows grazed 8 h or longer and farmers were willing to cooperate during data collection. On these farms the ambient temperature and the relative humidity were measured continuously in 1-hour intervals by using data loggers (Testo Datenlogger, Testo AG, Lenzkirch, Germany) during summer 2019 inside the barn and outside (in front of the barn under the roof). Furthermore, the farmers were asked to record daily when they kept their dairy cows inside the barn during the day to see how this factor would affect barn climate.

#### 2.1.2 Data processing

For each of the 19 Swiss dairy farms, data from 18 June to 10 September 2019 were considered. From this sample, days with a daily outside mean ambient temperature of at least 15 °C across all the dairy farms were selected, resulting in a total of 80 days. Data were subdivided in days with daily outside mean ambient temperature between 15 and 22 °C (*n* = 51, ambient temperature with a similar range as in the following experimental study) and days with daily outside mean ambient temperature above 22 °C (*n* = 29). As the barns could be expected to heat up because of the cows’ dissipation of body heat, we also categorised if the cows had been inside or not between 1000 and 1800 h on these days.

### 2.2 Experimental study

#### 2.2.1 Animals, experimental setup and grazing management

All experimental procedures were approved (No. 2018_04_FR) by the Committee of Animal Experiments of the Canton of Fribourg (Switzerland) and were in accordance with the Swiss guidelines for animal welfare. Data collection took place during the summer periods of 2018 (6 June to 7 September) and 2019 (15 June to 1 September) on the experimental farm of Agroscope in Posieux, Switzerland (46°46.01′N, 7°6.03′E; 676 m above sea level). Data collection was performed on a total of 38 dairy cows (51 % Holstein and 49 % Red-Holstein). As previously described in Pontiggia et al. (2023), 24 dairy cows were enrolled in each summer.

The present experimental study considers part of the data from a larger study where data were collected in 12 experimental periods of 4–6 days (six experimental periods in 2018 and six experimental periods in 2019). The first 2–3 days of each experimental period were used to investigate the individuals’ heat stress by physiological and behavioural indicators (Pontiggia et al., 2023, 2024) under full-time grazing conditions. For the present study, only the last 2–3 days of each experimental period were analysed. To protect the animals on pasture from severe heat stress, an experimental period was terminated prematurely if ambient temperature exceeded 25 °C at 1100 h on a given day; all cows were then brought into the free-stall barn until the evening milking (applies to 1 day of experimental period 11 and 1 day of experimental period 12).

In each year, the herd was divided into two groups of 12 animals that were comparable in traits known to influence heat stress susceptibility (milk production level, body weight, coat colour (black/red vs. white) and lactation stage). Within these two groups, cow pairs were created by matching animals that differed as much as possible in these traits, and these paired cows always grazed together throughout the duration of a summer. Two pairs each were combined to form six experimental groups of four animals.

During the last 2–3 days of each experimental period, grazing cows were subjected to two treatment conditions. Half of the cows (three experimental groups) stayed on pasture, and grazing was only interrupted for milking (from 0430 to 0730 h and from 1500 to 1730 h). The other half (three experimental groups) were brought in the barn during the hottest time of day (from 1130 h until the afternoon milking) and then released on pasture again. They were offered hay (mean absorbable protein at the duodenum: 64 ± 6.4 g/kg of dry matter; mean net energy for lactation: 4.3 ± 0.33 MJ/kg of dry matter) for ad libitum intake at the feeding fence. After each experimental period, treatments were switched to create a cross-over design. The composition of pairs within the experimental groups changed randomly every cross-over.

After morning and afternoon milking, the cows were offered an energy-enhanced concentrate feed according to their current milk yield (Agroscope, 2018). The concentrate contained (g/kg): maize grain, 440; wheat grain, 220; barley grain, 110; maize gluten, 90; mixed fat, 30; molasses, 20; CaCO_3_, 33; NaCl, 30; and trace elements–vitamin mix, 27. Non-iodised cattle salt was provided ad libitum on pasture (Pontiggia et al., 2023). All cows always had access to water on pasture (LA BUVETTE Lac, Tournes, France) and in the barn (SUEVIA HAIGES GmbH, Kirchheim am Neckar, Germany). Shading was not provided on pasture, and fans or sprinklers were not turned on inside the barn and milking parlour. Cows in heat were removed from the herd during the experimental period (two cows for two experimental days in two experimental periods). Experimental groups grazed in separate adjacent paddocks using a set stocking system. Paddock size varied between 1.0 and 1.3 ha and was adapted over the grazing period based on the current herbage growth.

#### 2.2.2 Milk yield and composition

Cows were milked in a milking parlour with individual milk yield recording (Pulsameter 2, SAC, Kolding, Denmark). Milk was sampled from every cow once daily at each afternoon milking. For later analysis of fat, protein and lactose, samples were conserved using Broad-Spectrum Microtabs II (Gerber Instruments AG, Effretikon, Switzerland) and stored at 5 °C.

#### 2.2.3 Climate data

As previously described in Pontiggia et al. (2023), ambient temperature in °C, relative humidity in %, wind speed in m/s and solar radiation in W/m^2^ were recorded every minute by using a mobile weather station (Onset, Bourne, MA) set up at the pasture site. Inside the barn, ambient temperature and relative humidity were recorded every 10 min by using two data loggers (Testo Datenlogger, Testo AG, Lenzkirch, Germany), which were located at heights of approximately 2 m. Recorded data were then averaged per time point. The wind speed inside the barn was recorded every minute during experimental periods 7–10 by using an anemometer (Strömungssensor SS 20.501, SCHMIDT® Technology, St. Georgen, Germany) located at a height of about 2 m. Mean wind speed inside the barn between 1200 and 1430 h was 0.23 m/s (± 0.03), and this mean was used as constant for further calculations. Solar radiation was set to zero in the barn. Climate data were used to calculate the CCI on pasture and inside the barn, which reflects the perceived ambient temperature in °C and provides an adjustment to ambient temperature for relative humidity, wind speed and solar radiation (Mader et al., 2010, 2011).

#### 2.2.4 Body temperature

Body temperature was measured by vaginal temperature and reticular temperature. The vaginal temperature of each cow was recorded continuously every 10 min with a microprocessor temperature logger (DST micro-T logger, Star-Oddi, Garðabær, Iceland; Pontiggia et al., 2023). The logger was secured to a progesterone-free, modified vaginal controlled internal drug-release device (Eazi-Breed CIDR, Zoetis, Parsippany, USA; length 13.5 cm, wingspan 15.0 cm) and inserted approximately 30 cm into the vaginal cavity at the start of each experimental period. The reticular temperature of each cow was recorded continuously every 10 min with a temperature sensor bolus (smaXtec, Graz, Austria; Pontiggia et al., 2024). At the start of the experiment, the boli were orally administered to each cow using an applicator, ensuring they were swallowed into the reticulum via the oesophagus.

Some data of the vaginal temperature were missing (9.0 %) because animals lost the loggers. Furthermore, values of vaginal temperature below 37.3 and above 40.4 °C (<0.01 %) were considered measurement errors according to Ammer et al. (2016a) and were excluded from the dataset. In addition, recordings of reticular temperature below 37 °C (5.4 %) were considered to be related to drinking events and were excluded from the dataset.

#### 2.2.5 Heart rate and respiration rate

Heart rate measurements in beats per minute were automatically recorded using the PolarTeam Pro system (Polar Electro Oy, Kempele, Finland; Pontiggia et al., 2023). Owing to technical reasons, 5.2 % of the heart rate data were missing.

The cows were directly observed by two trained observers who counted full breaths. Inter-observer reliability was 1.0 (Spearman correlation; *P* < 0.001). Cows were observed during two time windows of 2 h each (between 0900 and 1100 h on pasture and between 1230 and 1430 h on pasture or inside the barn) on every experimental day as previously described by Pontiggia et al. (2024). Each of the two observers was in charge of the observation of three experimental groups on every experimental day. The observer switched between the three experimental groups in intervals of 10 min. This led to four intervals per cow in each time window. In each interval, 10 consecutive full breaths were timed by using a stopwatch and the collected data was later converted to breaths per minute reflecting respiration rate (Tresoldi et al., 2016). Data could not be recorded between 0900 and 0930 h because cows were predominantly walking and grazing. The number of measurements per cow varied between 0 and 4 in a time window, resulting in 81 % missing values between 0930 and 1100 h and 46 % between 1230 and 1430 h, as only data from resting or standing cows could be collected.

#### 2.2.6 Behavioural traits

Each dairy cow was equipped with an accelerometer (MSR145 data logger, MSR Electronics GmbH, Seuzach, Switzerland), which measured the cow’s activity continuously (Pontiggia et al., 2023). The day before every experimental period, the accelerometer was attached at the metatarsus of the left hind leg, following the methods described by Weigele et al. (2018).

Based on these data, it was possible to determine lying duration and walking activity of each cow. The lying duration (hours) and the walking activity (*g*-force/hour) were calculated with R (version 4.1.2; R Core Team, 2021) according to Weigele et al. (2018).

The dairy cows were equipped with a RumiWatch recording device (Itin + Hoch GmbH, Liestal, Switzerland), which recorded their feeding behaviour continuously as previously described (Pontiggia et al., 2024). The feeding and ruminating durations (min) were calculated using the RumiWatch Converter (version 0.7.3.36) (Rombach et al., 2018). Owing to malfunctions of the logger, 14.3 % of the collected data were not included in the analysis.

#### 2.2.7 Statistical analyses

Statistical analyses were conducted with R software (version 4.2.0; R Core Team, 2022). For analysis, data of a total of 32 days were assigned to 38 cows with an unbalanced number of days per cow. The CCI in the barn and on pasture were compared in two separate paired *t*-tests between 0830 and 1100 h and between 1200 and 1430 h. The same comparison was made for ambient temperature.

The effect of treatment (cows kept on pasture or inside the barn) was investigated by using the lmer function for calculating linear mixed-effects models (lme4 package; Bates et al., 2015). Residuals of the calculated models were plotted and visually inspected for normal distribution and homoscedasticity. All variables were acceptable, and data were not subjected to any transformation for the analysis. To explore whether vaginal temperature, reticular temperature, heart rate and respiration rate differed between treatments, two separate linear mixed-effects regression models for the morning (0830–1100 h; except 0930–1100 h for respiration rate) and for the afternoon (1200–1430 h; except 1230–1400 h for respiration rate) were calculated. This approach enabled us to describe the course of these outcome variables over time (for the morning and afternoon separately) and its interaction with treatment. In all models, treatment was inserted as explanatory variable as well as time of sampling and their interaction. Time of sampling was estimated using natural splines (splines package; R Core Team 2022). Animal identity within experimental group identity, within day, within experimental period were inserted as nested random effects in all models. Additionally, the experimental group identity was added as a crossed random intercept. The *P*-values for the fixed effects were obtained using the mixed function (afex package; Singmann et al., 2024).

To explore whether lying duration, feeding duration, ruminating duration and walking activity were different between treatments, one linear mixed-effects regression model was specified for every outcome variable. In all models, treatment was inserted as explanatory variable, as well as time of sampling (coded as two-level factorial variable, morning or afternoon) and their interaction. Animal identity within experimental group identity, within day, within experimental period were inserted as nested random effects in all models. Additionally, the experimental group identity was added as a crossed random intercept. In all linear mixed-effects regression models, the *P*-values for the fixed effects were obtained using the mixed function.

## 3 Results

### 3.1 Pilot study: Climate conditions in barns with pasture-based milk production

Of the 19 farms, 41 % had a tie-stall system and 59 % loose housing. On seven farms the roof was insulated, on three farms the roof was not insulated, and on nine farms the state of roof insulation was not clearly established. On 50 % of the farms, ventilators or sprinklers were present (but we had no reliable information when they were used). On the 80 days measured, the average ambient temperature outside was higher than the average ambient temperature inside on 17 % of the days on farms with non-insulated roofs. On the other farms this was the case on 91 % of the days.

**Fig. 1:**
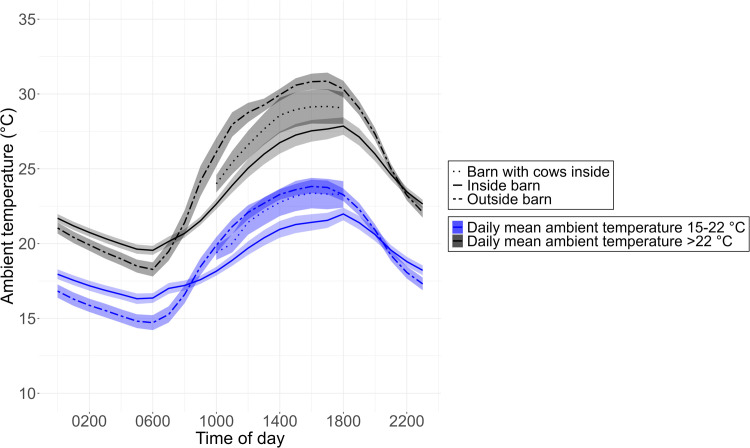
Distribution (mean and SE) of the ambient temperature ( °C) over the course of the day inside and outside the barn collected on 80 days on 19 farms during summer 2019 for the pilot study. Data are presented separately for days with a mean daily ambient temperature between 15 and 22 °C (*n* = 51) and for days with a mean daily ambient temperature above 22 °C (*n* = 29). The dotted lines represent the ambient temperature in the barns on days when the cows were inside from 1000 to 1800 h (data from nine barns on 51 and 29 days, respectively).

Between 1000 and 1800 h, cows were kept inside the barn on nine of the farms (mean ± SD) for 53 ± 25 % of the days with daily outside mean ambient temperature between 15 and 22 °C and for 79 ± 27 % of the days with daily outside mean ambient temperature above 22 °C. The ambient temperature inside the barn and outside showed a similar circadian rhythm, with low values during the night and constantly increasing values from 0800 until 1800 h (Fig. 1). On days when the daily outside mean ambient temperature was between 15 and 22 °C, the mean ambient temperature (± SD) from 1000 to 1800 h was 22.6 °C (± 2.79) outside and 20.5 °C (± 1.98) inside. If the barns were used by the cows in this time, the mean ambient temperature inside was 21.9 °C (± 2.28). On days when the daily outside mean ambient temperature was above 22 °C, the mean ambient temperature from 1000 to 1800 h was 29.4 °C (± 2.53) outside and 26.1 °C (± 2.16) inside. If the barns were used by the cows in this time, the mean ambient temperature inside was 27.2 °C (± 1.98).

### 3.2 Experimental study

#### 3.2.1 Climate conditions in the barn and on pasture

**Fig. 2:**
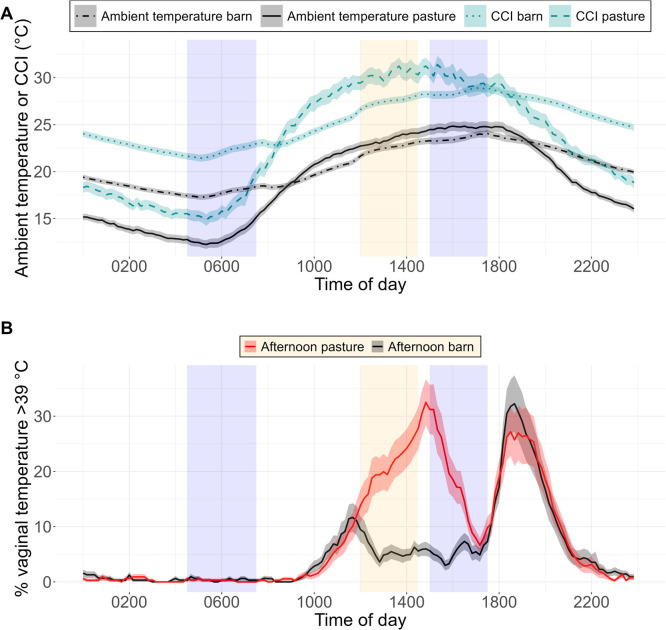
Distribution (mean and SE of 32 experimental days) of the comprehensive climate index (CCI, °C) and the ambient temperature ( °C) (A) and distribution of the cows’ vaginal temperature recordings greater than 39 °C (B) during the course of the day on pasture or inside the barn. On each day, half of the cows were kept inside the barn during the afternoon (1200 to 1430 h; orange area) while the other half stayed on pasture. Cows grazed full time except for milking (0430 to 0730 h and 1500 to 1730 h; blue areas).

Daily values for the CCI and its components ambient temperature, relative humidity, solar radiation and wind speed are summarised in Table 1. The CCI and the ambient temperature showed a similar daily variation, with low values during the night and constantly increasing values from the morning until the cows entered the barn for milking in the afternoon (Fig. 2A). The CCI fluctuated more on pasture than in the barn during the day. Over 24 h, the mean CCI on pasture was 23.6 ± 2.74 °C whereas that inside the barn was 25.3 ± 1.68 °C. On 84 % of the experimental days, the difference between the mean CCI on pasture and the mean CCI inside the barn from 1200 to 1430 h was positive. Across all experimental days, the mean CCI from 1200 to 1430 h was higher on pasture (30.3 ± 4.45 °C) than inside the barn (27.5 ± 1.97 °C) (*P* < 0.001). Also, the mean ambient temperature from 1200 to 1430 h was higher on pasture (23.5 ± 2.74 °C) than inside the barn (22.5 ± 1.68 °C) (*P* < 0.01).

**Table 1:** Daily values (mean and SD, minimum [min.], maximum [max.]) of the comprehensive climate index (CCI, °C) and its components: ambient temperature ( °C), relative humidity ( %), wind speed (m/s) and solar radiation (W/m^2^) assessed in 12 experimental periods in the morning, the afternoon and over 24 h on pasture and in the barn. Cows grazed full time except for milking (0430 to 0730 h and 1500 to 1730 h). All cows were on pasture during the morning (0830 to 1100 h). Half of the cows were kept in the barn during the afternoon (1200 to 1430 h) while the other half stayed on pasture during this time.

	Pasture				Barn			
Parameter	Mean	SD	Min.	Max.	Mean	SD	Min.	Max.
Morning	All cows on pasture				No cow in the barn			
CCI ( °C)	26.4	4.04	18.7	33.0	24.0	1.90	19.1	28.8
Ambient temperature ( °C)	20.1	2.30	15.6	24.0	19.4	1.53	15.4	23.3
Relative humidity ( %)	71.3	10.3	37.5	85.8	68.0	6.56	50.9	77.8
Wind speed (m/s)	1.07	1.08	0	4.14	0.17	0.07	0.10	0.32
Solar radiation (W/m^2^)	505	162	140	800	–	–	–	–
Afternoon	Half of the cows on pasture				Half of the cows in the barn			
CCI ( °C)	30.3	4.45	19.8	36.6	27.5	1.97	23.4	30.9
Ambient temperature ( °C)	23.5	2.70	16.6	29.3	22.5	1.68	19.0	25.4
Relative humidity ( %)	59.6	12.0	32.1	94.3	59.3	8.76	39.7	81.4
Wind speed (m/s)	1.69	1.31	0.36	6.40	0.23	0.03	0.18	0.28
Solar radiation (W/m^2^)	704	214	64.0	963	–	–	–	–
24 h								
CCI ( °C)	23.6	2.74	16.9	28.5	25.3	1.68	21.0	28.3
Ambient temperature ( °C)	19.1	1.78	14.9	22.3	20.7	1.35	17.0	23.0
Relative humidity ( %)	72.5	9.15	52.4	88.3	62.8	7.50	47.9	76.3
Wind speed (m/s)	0.95	0.81	0.18	3.73	0.16	0.02	0.13	0.20
Solar radiation (W/m^2^)	271	83.3	87.4	479	–	–	–	–

#### 3.2.2 Hay consumption, milk yield and composition

The cows consumed a mean of 1.8 ± 0.78 kg dry matter of hay when kept in the barn. The mean daily milk yield (± SD) for cows on pasture and those going into the barn in the afternoon was 28.4 kg (± 4.93) and 28.8 kg (± 4.72), respectively. The concentration of milk fat was 3.90 % (± 0.44) vs 3.93 % (± 0.43), that of lactose 4.61 % (± 0.14) vs 4.63 % (± 0.14) and that of protein 3.23 % (± 0.18) vs 3.22 % (± 0.18).

#### 3.2.3 Effect of the cooling measure on physiological responses

The cows had very similar patterns in their daily distribution of vaginal temperature recordings above 39 °C except during the time the treatment was applied (Fig. 2B). During the night, the percentage of vaginal temperature recordings above 39 °C remained constant at about 2 % and lower. After 1000 h, the percentage constantly increased to about 10 %. Between 1130 and 1500 h, for cows on pasture the percentage further increased to about 30 %, then decreased after 1500 h to about 5 % at 1730 h when the animals where inside the barn for milking. At 1130 h, the percentage of vaginal temperature recordings above 39 °C decreased in cows that entered the barn at that time and remained constant at about 5 % from 1230 to 1730 h. After the afternoon milking, both groups showed again a similar increase with a second peak for vaginal temperature recordings above 39 °C of about 25 % or higher after 1800 h.

**Fig. 3:**
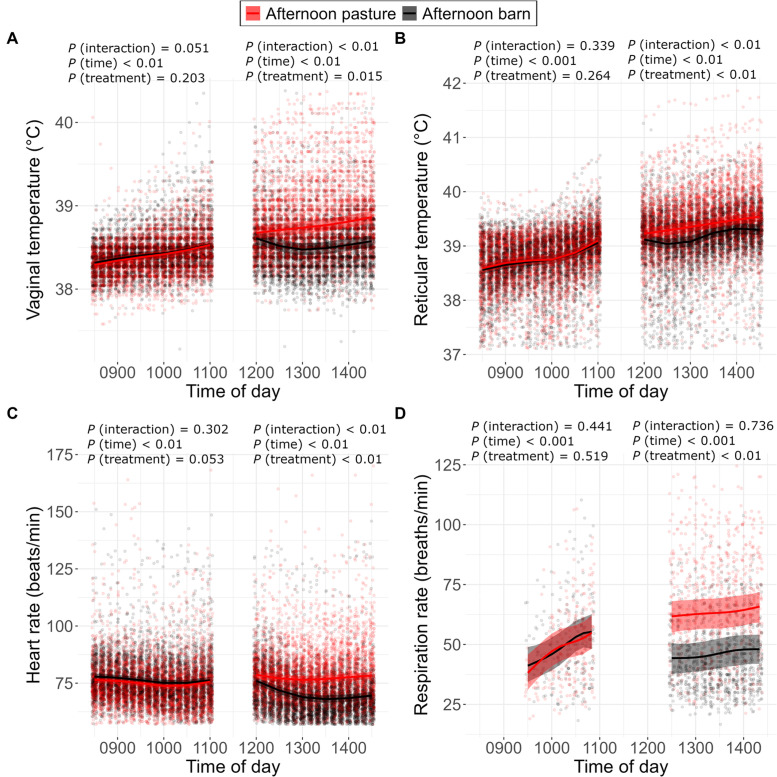
Distribution (estimate and confidence interval) of vaginal temperature ( °C) (A), reticular temperature ( °C) (B), heart rate (beats per minute) (C) and respiration rate (breaths per minute) (D) recorded during the morning (0830–1100 h; except 0930–1100 h for respiration rate) and during the afternoon (1200–1430 h; except 1230–1400 h for respiration rate) in individual dairy cows that were kept either on pasture or in the barn during the afternoon (1200 to 1430 h) on the experimental days (*n* = 32). The line and the shaded areas represent model estimates and 95 % confidence intervals, respectively.

In the morning, the cows that remained on pasture all day and those going into the barn in the afternoon displayed similar patterns of vaginal temperature (*P* < 0.01 time; *P* = 0.051 interaction; *P* = 0.203 treatment; Fig. 3A), reticular temperature (*P* < 0.001 time; *P* = 0.339 interaction; *P* = 0.264 treatment; Fig. 3B), heart rate (*P* < 0.01 time; *P* = 0.053 treatment; *P* = 0.302 interaction; Fig. 3C) and respiration rate (*P* < 0.001 time; *P* = 0.441 interaction; *P* = 0.519 treatment; Fig. 3D). The estimated change over time was from 38.3 [38.3, 38.4] to 38.5 [38.5, 38.6] °C (model estimate, 95 % confidence interval) for vaginal temperature, from 38.6 [38.5, 38.7] to 39.1 [39.0, 39.2] °C for reticular temperature, from 77.2 [75.8, 78.5] to 76.0 [74.6, 77.3] beats per minute for heart rate and from 39.7 [32.7, 47.3] to 55.1 [48.3, 62.4] breaths per minute for respiration rate.

In the afternoon, vaginal temperature (*P* < 0.01 interaction), reticular temperature (*P* < 0.01 interaction) and heart rate (*P* < 0.01 interaction) were estimated to develop differently in the two treatments. In cows on pasture, vaginal temperature increased over time from 38.7 [38.6, 38.8] to 38.9 [38.8, 38.9] °C, while it remained at 38.6 [38.5, 38.7] °C in cows inside the barn (*P* < 0.01 time) so that cows on pasture had a higher vaginal temperature than cows in the barn (*P* = 0.015 treatment). Reticular temperature was estimated to increase over time (*P* < 0.01 time) with higher levels for cows on pasture (from 39.2 [39.1, 39.3] to 39.5 [39.5, 39.6] °C) than for cows in the barn (from 39.1 [39.0, 39.2] to 39.3 [39.2, 39.4] °C; *P* < 0.01 treatment). In cows on pasture, heart rate was estimated to decrease over time from 76.0 [74.4, 77.6] to 69.6 [67.9, 71.2] beats per minute in cows in the barn, while it slightly decreased from 78.7 [77.0, 80.3] to 78.4 [76.8, 79.9] beats per minute in cows on pasture (*P* < 0.01 time), and cows on pasture had a higher heart rate than cows in the barn (*P* < 0.01 treatment). In the afternoon, respiration rate showed a similar increase over time in the two treatments (*P* < 0.001 time; *P* = 0.736 interaction) but cows on pasture (increase from 61.7 [55.1, 68.0] to 65.8 [59.3, 71.9] breaths per minute) had a higher respiration rate than cows in the barn (increase from 44.3 [37.8, 50.5] to 48.0 [41.9, 54.1] breaths per minute; *P* < 0.01 treatment).

#### 3.2.4 Effect of the cooling measure on behavioural responses

**Fig. 4:**
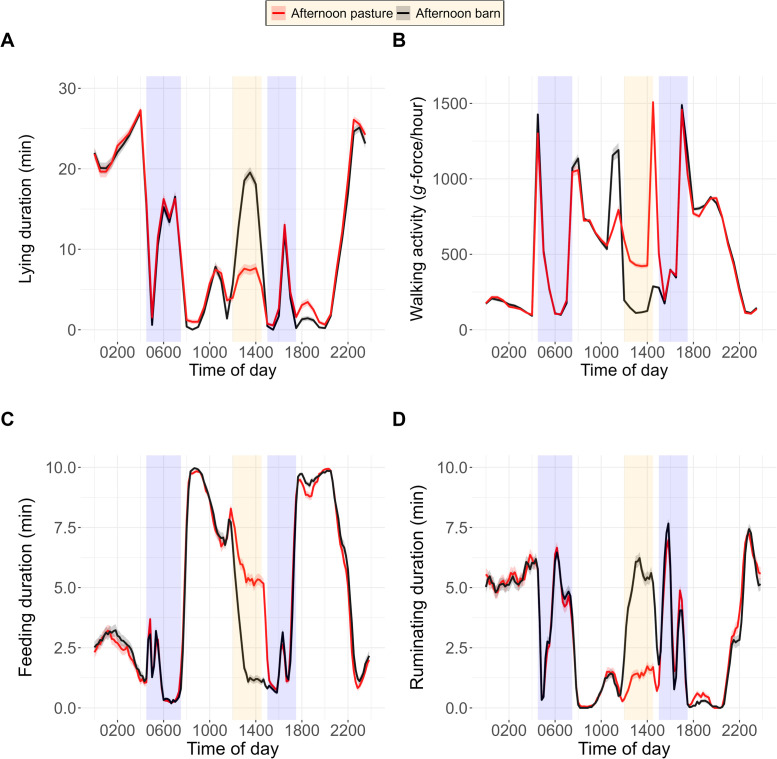
Distribution (mean and SE) of lying duration (min, for 30-min intervals) (A), walking activity (*g*-force/hour) (B), feeding duration (min, for 10-min intervals) and ruminating duration (min, for 10-min intervals) (D) during the course of the day. Dairy cows were kept either on pasture or in the barn during the afternoon (1200 to 1430 h; orange area) on the experimental days (*n* = 32). All cows were in the barn for milking from 0430 to 0730 h and from 1500 to 1730 h (blue areas).

**Fig. 5:**
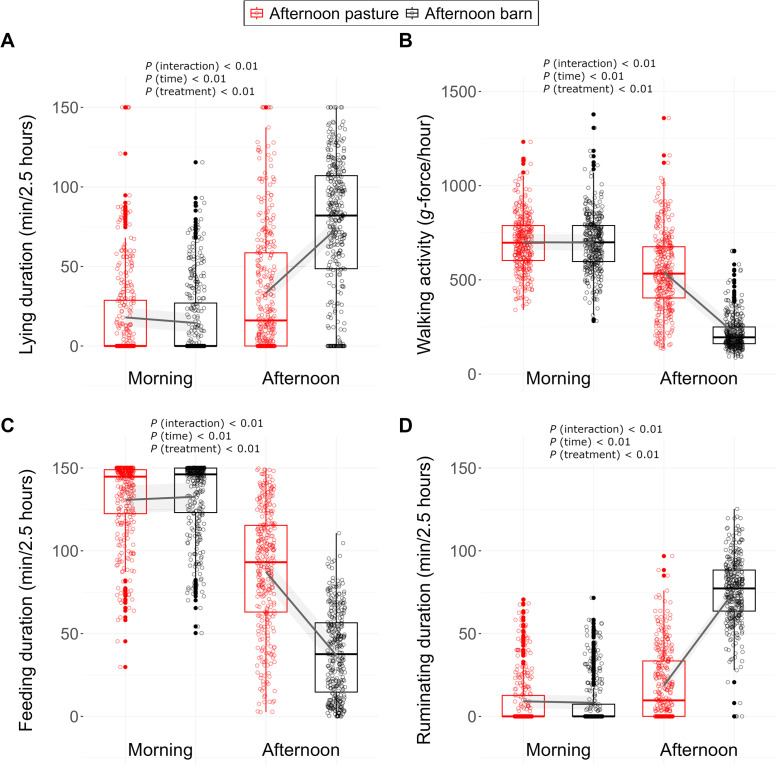
Lying duration (min/2.5 h) (A), walking activity (*g*-force/hour) (B), feeding duration (min/2.5 h) (C) and ruminating duration (min/2.5 h) (D) recorded between 0830 and 1100 h (morning) and between 1200 and 1430 h (afternoon) in grazing dairy cows that were kept either on pasture or in the barn between 1200 and 1430 h on the experimental days (*n* = 32). Dots represent raw data. Boxplots indicate the 25th percentile, the median and the 75th percentile. The line and the shaded areas represent model estimates and 95 % confidence intervals, respectively.

The time cows spent with lying, walking activity, feeding and ruminating showed a circadian rhythm (Fig. 4A–D). The rhythm of feeding and walking activity was opposite to the patterns of ruminating and lying. Treatments had very similar patterns except during the time when the two treatment conditions were applied. Lying duration, walking activity, feeding duration and ruminating duration changed differently from the morning to the afternoon in the two treatments (for all outcome variables *P* < 0.01 interaction; *P* < 0.01 time; *P* < 0.01 treatment) (Fig. 5A–D). In the morning, the cows that remained on pasture all day and those going into the barn in the afternoon had similar levels of lying duration (18.0 [12.3, 24.0] vs 14.5 [8.59, 20.3] min), walking activity (700 [659, 744] vs 698 [655, 742] *g*-force/hour), feeding duration (131 [122, 138] vs 133 [124, 141] min) and ruminating duration (9.07 [4.90, 13.2] vs. 8.23 [4.15, 12.5] min). In the afternoon, compared with the cows on pasture, the cows in the barn lay more (+41.3 min), walked less (−325 g-force/hour), ate less (−50.4 min) and ruminated more (+57.1 min).

## 4 Discussion

In the present study, we assessed if bringing grazing dairy cows inside the barn during the hottest time of day is an effective measure to mitigate their heat stress under moderate heat load conditions. To effectively reduce heat stress, the climate conditions in the barn need to be cooler than those on pasture. Based on the results of the present pilot study on Swiss dairy farms practicing grazing, relevantly cooler conditions can be expected between 1000 and 1800 h inside the barn than outside. In the present experimental study, under similar climate conditions and with a similar heat load difference inside the barn and outside, we could show that physiological responses of dairy cows to heat stress were less pronounced when animals were kept inside the barn during the afternoon than when they remained on pasture during this time.

### 4.1 Climate conditions in the barn and on pasture

In the present study, we aimed at investigating to what extent an already practiced measure in dairy farming, namely bringing the cows inside the barn during the hottest time of day on hot summer days (Thanner et al., 2014), can mitigate heat stress under moderate conditions. Although the 19 farms investigated in the pilot study differed largely in terms of housing system, barn construction and equipment (e.g. availability of ventilators or sprinklers), for most farms and on most days the barns were indeed cooler than the outside temperature during the day. Even if this difference decreased when cows were kept inside, the ambient temperature inside could still be expected to be cooler. The lack of roof insulation might explain why three barns were not cooler than outside during the day. It was not possible to measure the CCI on the farms, but it can be expected that the difference in CCI would have been bigger than the difference in ambient temperature, as the cows were protected from solar radiation. At least for barns like those in our experimental study, a lower heat load inside the barn can be reached during the hottest time of day under the climate conditions investigated.

In the experimental study, the climate conditions on pasture with a daily mean ambient temperature of 19 °C were comparable to those reported in similar studies on heat stress in moderate climates (Kendall et al., 2006; Tucker et al., 2008). The differences in ambient temperature from 1200 to 1430 h between barn and pasture we measured were similar to those in the pilot study when cows were inside the barn (on average −1 °C ambient temperature). Therefore, we can speculate that the calculated differences in CCI (on average −3.0 °C CCI) in the experimental study indicate that the barn protected the dairy cows from intense solar radiation rather than providing a lower ambient temperature.

### 4.2 Assessment of heat stress

Previous studies have shown that recordings of core body temperature above 39.0 °C are indicative of a state of mild hyperthermia, which has been linked to impaired milk production and fertility in dairy cows (Kadokawa et al., 2012). At night, vaginal temperature recordings above 39 °C were very rare (1–2 %), suggesting that all cows were able to recover from the heat load experienced the day before. During the day, a steady increase in vaginal temperature recordings above 39 °C in parallel with an increasing CCI during the day indicates that cows reacted to the heat load. The long (350 to 1500 m) and ascending distances walked to reach their paddock after afternoon milking in addition to the heat load accumulating over the day and a rather high CCI at this time of day may explain the peak in vaginal temperature values above 39 °C at about 1830 h in both treatment groups. After morning milking at about 0800 h (when the cows reached their paddock), climate conditions apparently were not yet such as to evoke such a peak in vaginal temperature.

During the morning, when all cows were on pasture, a difference in treatment groups could not be detected in their physiological and behavioural indicators and it can be assumed that cows in both treatment groups had the same baseline. We can therefore argue that the cows in our study were repeatedly exposed to a short-term moderate heat load (Pontiggia et al., 2023) and that differences occurring between treatments in the afternoon were caused by the differing heat load to which the cows were exposed.

### 4.3 Effects of bringing dairy cows in the barn during the afternoon on physiological and behaviour responses

The gold standard for assessing heat stress is the rise in core body temperature (Ammer et al., 2016b; Veissier et al., 2018). It is well known that vaginal temperature is a reliable measure of core body temperature (Idris et al., 2021). Despite the presence of reticulorumen environmental perturbations (e.g. by water and feed ingestion), reticular temperature can be considered as a proxy for assessing core body temperature and can be used as an alternative suitable indicator for assessing heat stress in dairy cows (Ammer et al., 2016b; Pontiggia et al., 2024). Results of the present study suggest that increase in vaginal and reticular temperatures of dairy cows on pasture were due to the increase in heat load. During the afternoon, cows kept inside the barn showed mean vaginal and reticular temperatures of about 0.3 °C lower than the cows on pasture. This difference indicates that bringing dairy cows inside the barn during the hottest time of day is an effective cooling measure. While the vaginal temperature remained constant during the afternoon in cows kept inside, their reticular temperature increased constantly during this time. This could be attributed to their increased ruminating activity during the same period, which might have increased the metabolic heat production (Kadzere et al., 2002).

Previous studies have indicated that cows can improve heat dissipation by rising their respiration rate and heart rate (Kadzere et al., 2002; Brosh, 2007). In addition to vaginal and reticular temperature, changes in respiration rate and heart rate in the present study suggested that cows being inside during the hottest time of day were less heat stressed than cows remaining on pasture. During the afternoon, cows in the barn showed a mean respiration rate of about 21.6 breaths per minute lower than that of cows on pasture. Furthermore, respiration rates which have previously been classified as elevated (>48 breaths per minute) (Li et al., 2020) were rarely observed in the cows inside the barn.

The heart rate of cows on pasture was rather stable over time, suggesting that its response to increasing heat load was not as sensible as that of core body temperature. During the afternoon, the mean heart rate of cows kept inside was about 7.0 beats per minute lower than that of cows on pasture. Furthermore, it was even 4.8 beats per minute lower than the heart rate measured on pasture during the morning (where all cows experienced similar climate conditions). These results suggest that not only the lower heat load but also other factors influenced the heart rate of cows in the barn, such as the reduced walking activity and increased lying duration.

In addition to physiological changes, several behavioural adaptations can be expected in dairy cows under hot conditions in the attempt to increase their heat dissipation or reduce their metabolic heat production. In the present study, when inside the barn, cows showed longer lying (+124 %) and ruminating durations (+307 %) and lower walking activity (−60 %) and feeding duration (−58 %) than on pasture. According to previous studies performed in free-stall barns where cows were also subjected to varying heat load, we would expect a difference in ruminating and lying durations of about 5–10 % (Herbut & Angrecka, 2018; Müschner-Siemens et al., 2020). Moreover, previous studies reported longer lying times in tie-stall and free-stall systems than in pasture-based systems (Tucker et al., 2021). Therefore, the difference in these behaviours observed in our study cannot be attributed exclusively to the differing climate conditions inside the barn and on pasture but rather to the different housing conditions (e.g. space, cubicles, access to hay).

In the present study the interpretation of feeding behaviour data was limited by the inability to measure individual feed intake and by the differences between treatments in the feed provided. Therefore, differences between treatments in feeding duration may simply arise because the same amount of dry matter was eaten faster in form of hay than grass by grazing (Graf et al., 2005). The cows may have consumed less dry matter in the barn than on pasture, as the amount of hay consumed in the barn was very low (1.8 ± 0.78 kg dry matter). Furthermore, the hay had a lower nutritional value than grass (Pontiggia et al., 2023). In fact, we could observe a slight increase in feeding upon return to pasture (at about 1800 h) in cows that had been inside the barn during the hottest time of day. These results may suggest that the cows that had been inside the barn were compensating for the reduced energy intake. Moreover, although being inside the barn during the hottest time of day reduced their grazing time, the cows may have compensated the reduced access time to pasture by increasing their feed intake per minute (Kennedy et al., 2009). Further studies are needed to assess if the restricted grazing time will affect the feed intake in the long term and if dairy cows can compensate for it by changing their circadian rhythm. The treatment duration in the present study was limited to 12 experimental periods of 2 to 3 consecutive days, conducted over two years (18 days per year). This relatively short and discontinuous timeframe may have limited the detection of cumulative or delayed effects on production-related parameters such as milk yield and composition, which might only appear after prolonged application of the treatment (Van Laer et al., 2015a). Although milk yield and composition were similar between treatment groups — suggesting no substantial differences in feed intake in the short term — these findings should be interpreted cautiously. It remains unclear whether prolonged application of the treatment, could lead to changes in feeding behaviour, milk yield and composition.

It can therefore be assumed that under the present experimental conditions, bringing grazing dairy cows inside during the hottest time of hot summer days did not adversely influence animal behaviour but was able to relevantly lower their physiological heat stress response. Previous studies conducted under similar moderate climate conditions have shown that providing dairy cows with access to shade on pasture was effective in reducing mean vaginal temperature by about 0.1 °C (Kendall et al., 2006; Schütz et al., 2010). In the present study, dairy cows in the barn showed an average vaginal temperature that was about 0.3 °C lower than on pasture. When the ambient temperature in the barn is lower than on pasture, bringing dairy cows in the barn should therefore be an effective measure to reduce the heat stress for grazing dairy cows. It might be more efficient than the provision of shade on pasture as further measures could be applied such as increasing the wind speed (e.g. by fans) and reducing ambient temperatures (e.g. by sprinklers). Nonetheless, the practical feasibility of this mitigating strategy is largely context dependent. The distance to the barn, the availability of labour and the aim of maximising feed intake through grazing are among the factors that influence its applicability. This highlights the importance of tailoring heat abatement measures to specific farm conditions.

## 5 Conclusion

According to our pilot study, under moderate climate conditions relevantly cooler conditions can be reached during the day inside the barn than outside in farming practice. In our experiment, with a difference in CCI of 3 °C, cows inside the barn had lower body temperature, heart rate and respiration rate than cows on pasture and did not relevantly change their behaviour. Therefore, bringing grazing dairy cows inside during the hottest time of the day can be recommended to mitigate heat stress, especially when shade cannot be provided on pastures. Longer-term studies are needed to evaluate whether continued application of this mitigating strategy throughout an entire summer season would lead to negative impacts on feed intake or production-related parameters, and to assess any potential trade-offs between reduced heat stress and changes in feed intake or productivity.

## Ethics approval

All experimental procedures were approved (2018_04_FR) by the Committee of Animal Experiments of the Canton of Fribourg (Switzerland) and were in accordance with the Swiss guidelines for animal welfare.

## Data and model availability statement

None of the data were deposited in an official repository, but they are available upon request.

## Declaration of generative AI and AI-assisted technologies in the writing process

The authors did not use any artificial intelligence assisted technologies in the writing process.

## Funding sources

This work was supported by the Swiss Federal Food Safety and Veterinary Office (Project No 2.18.03, Bern, Switzerland), the Swiss Federal Office for Agriculture (Bern, Switzerland) and the Foundation Sur-la-Croix (Basel, Switzerland).

## CRediT authorship contribution statement

**Alice Pontiggia:** Writing – review & editing, Writing – original draft, Visualization, Project administration, Methodology, Investigation, Formal analysis, Data curation. **Mirjam Holinger:** Writing – review & editing, Formal analysis. **Andreas Münger:** Writing – review & editing, Methodology, Investigation. **Stefanie Ammer:** Writing – review & editing, Conceptualization. **Frigga Dohme-Meier:** Writing – review & editing, Supervision, Resources, Project administration, Methodology, Funding acquisition, Conceptualization. **Nina Maria Keil:** Writing – review & editing, Supervision, Resources, Project administration, Methodology, Funding acquisition, Conceptualization.

## Declaration of competing interest

The authors declare that they have no conflict of interest.
